# The National Pension Fund Established

**Published:** 1888-02-11

**Authors:** 


### The Hospital

A Weekly Institutional Journal of

**Science, Medicine, and Philanthropy.**

*For Hospitals, Asylums, Medical Practitioners, Students, Nurses, Families, Parishes, Congregations, and the Charitable Public.*

Vol. III. No. 72.] [February 11, 1888.

### The National Pension Fund Established.

The National Pension Fund for Nurses and other Hospital Officials, which was first a dream and then a possibility, has become an established and concrete fact. All practical men and women whom it may nearly or remotely concern must now give to it that kind of earnest and careful thought which actual and tangible things demand. So long as it was a mere idea it could be treated by the critic in any way the mood of the moment dictated. He could think it a good suggestion and express a pious hope that it would one day become a working reality; or he could scoff at it as the fleeting dream of an unpractical enthusiast's brain. Those courses are no longer open either to friends or foes. The thing is here; here to be considered; here to be approved or condemned by the critic; here to be accepted or rejected by the nurse. A day of decision on all kinds of practical questions now and again comes to every man and woman, and on the decision made at the critical moment large future issues not infrequently depend. What we wish to emphasise and make clear is this, that in the case of people who have to look solely to their own exertions for the whole of life it is imperative that they learn to think intelligently and to act with decision for themselves. If they give their affairs into the keeping of other people, and acquire the habit of indecision, they will find sooner or later that whilst other people will have no hesitation in leading them into bogs and quicksands for their own convenience, those people have neither the power nor the will to get them out of the dangers again.

Fifteen hundred nurses have affirmed their conviction that a Pension Fund would be a good thing; and they have proved the strength of their convictions by declaring their readiness to join such a fund if established on a basis which they can approve. What are the criteria by which nurses and others should be guided in deciding whether to join the Pension Fund or not? The first criterion would naturally be this—the security of the Fund. If a nurse should decide to pay in an eighth of her income annually in order to secure an allowance of ten shillings a week in case of sickness, or a similar sum weekly for life in case of permanent disablement, or after reaching the age of sixty, will she be sure of obtaining all these benefits at the time she may need them? That is of course a fundamental question. The answer is that she will be sure to obtain them. The Fund is as secure as an Act of the British Parliament, and as the wisest and most successful English financiers can make it. The Council includes two directors of the Bank of England and a representative of each of the four great firms who have provided the deposit of twenty thousand pounds and placed it under Government control.

The next question a practical person would ask is, Are the benefits commensurate with the outlay? That is a question easily answered. Is it a great relief to persons who have no stored resources to feel sure that they can rely upon the means to secure a home and care in case of sickness, and that in the event of permanent disablement or when too old to work they can be sure of a sum which will be sufficient to meet all their simple needs? Is there one nurse in the United Kingdom, who has the sense to be a nurse, who will answer "No" to these questions? The National Pension Fund, which, in addition to the twenty thousand pounds deposited as security, will start with a Bonus Fund of several thousand pounds promised to Mr. Burdett as a free gift, is organised to secure to nurses a minimum sum of ten shillings a-week during sickness, or permanent disablement, or as an annuity for life after the attainment of sixty years of age. For this a premium of three pounds five shillings annually, or five shillings and sixpence a month will be paid by the nurse who starts at twenty-five years of age. "Well, but," asks the nurse, "can I not, by investing the money myself, or by joining some other society, obtain better results?" That is a plain and pertinent question, and the answer is equally pertinent and plain. By investing her money on her own account in the Post Office or elsewhere, a nurse cannot obtain anything like the results offered by the Pension Fund. The National Pension Fund is the only Fund now existing which offers pensions to nurses and other hospital officials. This society commences its operations with a deposit of twenty thousand pounds. It is not, therefore, a question of doing better elsewhere. There is no elsewhere. Here or nowhere is the provision which nurses can secure against sickness, disablement, and old age.

These are the two main points for a nurse's consideration—the security of the fund, and the advantages it offers. In both respects the fund has absolutely no rival anywhere; in both respects its value cannot be even called in question. There are certain subsidiary points also of great importance. Two of these are worthy of special note. The Fund will be the mutual society of Nurses and Hospital officials themselves, and they will have a share in its management. There is no demoralizing charity about it any more than there is about the colleges at Oxford and Cambridge, which owe their creation to the liberality of pious and generous founders. The Rothschilds, the Gibbs, the Hambros, and the Morgans have assumed the position of the generous founders towards the National Pension Fund, and many will follow their lead by contributing out of full and grateful hearts to the Bonus Fund for services rendered to themselves and their families. That is all. The nurses who join the Fund can, therefore, avail themselves of its advantages with a clear conscience. A second point is that it will tend constantly to grow in wealth and in its power to increase the amount of its pensions or to diminish the premiums paid by nurses for its benefits.

The National Pension Fund, strange to say, meets with decided opposition in certain quarters. To many, no doubt, this will appear incredible and almost incomprehensible. It is nevertheless true; and it is quite necessary to allude to the fact, because among practical people, especially among those who place large sums of money at the disposal of benevolent objects, results are looked for; and if results are not forthcoming the benevolent donors of money will promptly take back their offered help. If it should prove that the opponents of the National Pension Fund, who have nothing whatever to put in its place, should possess such influence with nurses and hospital officials as to prevent them availing themselves of its great and striking advantages before January 1st, 1890, the depositors of the twenty thousand pounds, who have retained the conditional right to do so, and the donors to the Bonus Fund will withdraw their princely aid. It is hardly conceivable that a body of fairly intelligent women like trained nurses will deliberately refuse such a sum as is already subscribed, and the infinitely larger sums which a fund started under such auspices would command in the future. Still more difficult is it to believe that any body of educated and conscientious men and women will dare to incur the responsibility and the public odium which would inevitably result if they were to succeed in keeping the nurses outside the Pension Fund. It may be taken as absolutely certain that no second opportunity of a like kind will ever be offered if those for whom the Fund is provided should show a complete incapacity to appreciate the noble generosity of those who are offering to help them at the present moment.

We put these issues plainly before all the parties directly interested, not because we really doubt their ultimate decision, but in order that they may see and know exactly how they stand in relation to this important question, and may act with the sense and decision befitting the greatness of the occasion. The present position of the National Pension Fund is the result of a long and arduous struggle—a struggle known only to a few. Its author is content to leave it now for the consideration of those whom it most nearly concerns. By the end of the present month the fullest information will be ready for distribution; and application should be made with as little delay as possible to the Secretary, 38, Old Jewry, E.C. It ought not to be too much to expect that in presence of the momentous practical issues involved, all personal and other trifling considerations will be put aside, and that the whole hospital world will unite in carrying to a successful issue one of the greatest and most benevolent schemes of modern times.

